# A Novel Geriatric Screening Tool in Older Patients with Cancer: The Korean Cancer Study Group Geriatric Score (KG)-7

**DOI:** 10.1371/journal.pone.0138304

**Published:** 2015-09-24

**Authors:** Jin Won Kim, Se-Hyun Kim, Yu Jung Kim, Keun-Wook Lee, Kwang-Il Kim, Jong Seok Lee, Cheol-Ho Kim, Jee Hyun Kim, Korean Cancer Study Group Geriatric Oncology Working Party

**Affiliations:** 1 Division of Hematology and Medical Oncology, Department of Internal Medicine, Seoul National University Bundang Hospital, Seoul National University College of Medicine, Seongnam, Republic of Korea; 2 Division of Geriatrics, Department of Internal Medicine, Seoul National University Bundang Hospital, Seoul National University College of Medicine, Seongnam, Republic of Korea; Banner Alzheimer's Institute, UNITED STATES

## Abstract

Geriatric assessment (GA) is resource-consuming, necessitating screening tools to select appropriate patients who need full GA. The objective of this study is to design a novel geriatric screening tool with easy-to-answer questions and high performance objectively selected from a large dataset to represent each domain of GA. A development cohort was constructed from 1284 patients who received GA from May 2004 to April 2007. Items representing each domain of functional status, cognitive function, nutritional status, and psychological status in GA were selected according to sensitivity (SE) and specificity (SP). Of the selected items, the final questions were chosen by a panel of oncologists and geriatricians to encompass most domains evenly and also by feasibility and use with cancer patients. The selected screening questions were validated in a separate cohort of 98 cancer patients. The novel screening tool, the Korean Cancer Study Group Geriatric Score (KG)-7, consisted of 7 items representing each domain of GA. KG-7 had a maximal area under the curve (AUC) of 0.93 (95% confidence interval (CI) 0.92−0.95) in the prediction of abnormal GA, which was higher than that of G-8 (0.87, 95% CI 0.85–0.89) within the development cohort. The cut-off value was decided at ≤ 5 points, with a SE of 95.0%, SP of 59.2%, positive predictive value (PPV) of 85.3%, and negative predictive value (NPV) of 82.6%. In the validation cohort, the AUC was 0.82 (95% CI 0.73−0.90), and the SE, SP, PPV, and NPV were 89.5%, 48.6%, 77.3%, and 75.0%, respectively. Furthermore, patients with higher KG-7 scores showed significantly longer overall survival (OS) in the development and validation cohorts. In conclusions, the KG-7 showed high SE and NPV to predict abnormal GA. The KG-7 also predicted OS. Given the results of our studies, the KG-7 could be used effectively in countries with high patient burden and low resources to select patients in need of full GA and intervention.

## Introduction

Geriatric assessment (GA) was developed to detect disabilities and geriatric conditions that can contribute to frailty, including functional, psychological, social, and nutritional deficits [1, 2]. GA is useful to guide treatment decisions in older patients who have diminished physiologic reserves [3]. In geriatric oncology, GA has been shown to predict the surgical complications [4], chemotherapy toxicity [5, 6], cancer patient survival [7, 8], and cancer treatment tolerance [9]. An important role of GA in oncology is to identify fit older patients who can receive standard cancer treatments [10]. However, full GA consists of several domains with many items related to medical, functional, neuropsychiatric, and social assessments [11]. Full GA is time-consuming and labor-intensive. Therefore, a 2-stepped approach using screening tools has been suggested recently to identify patients who should receive full GA to tailor treatment plans [12].

Several screening tools including the Geriatric 8 (G-8), Groningen Frailty Index (GFI), Abbreviated Comprehensive Geriatric Assessment (aCGA), Triage Risk Screening Tool (TRST), and Vulnerable Elders Survey-13 (VES-13) have been evaluated and validated in older patients with cancer [12–15]. However, some screening tools were produced for a different purpose, such as the VES-13, which identifies vulnerable older people in the community, and the TRST, which predicts repeat emergency department visits [16, 17]. Not all domains were included evenly across the screening tools [18, 19]. The respective sensitivity (SE), specificity (SP), positive predictive value (PPV), and negative predictive value (NPV) of each screening tool showed insufficient discriminative power to select patients for further assessment [19, 20]. In previous systematic review for performance of screening tool, the median SE and SP was 87% and 61%, respectively in G-8, 92% and 47% in TRST (patient considered frail if one or more impairments present), 68% and 78%, in VES-1, and 51% and 97% in aCGA. However, even in case of the highest SE, the NPV was only roughly 60% [19]. In particular, a screening tool suitable for high-burden oncology clinics with limited manpower is needed.

Consequently, we aimed to produce a more performant screening tool with high SE and NPV for older patients with cancer using a large dataset of individuals who received full GA and encompassing items across most domains evenly with high SE. We validated our novel screening tool in a retrospective cohort of older patients with cancer. The performance of KG-7 for predicting abnormal GA was compared with that of G-8. We also assessed the prognostic value of our screening tool in terms of overall survival (OS).

## Materials and Methods

### Development cohort

A development cohort (*N* = 1284) was constructed from all consecutive patients who received full GA in a single academic hospital from May 2004 to April 2007. GA was performed by a multidisciplinary team of geriatric nurses, nutritionists, and pharmacists. Patients in the development cohort had various diseases including 85 patients with a past medical history of cancer (6.6%) and 72 patients with current cancer (5.6%). GA results were retrieved from electronic medical records.

### Validation cohort

A validation cohort (*N* = 98) was derived from an independent retrospective cohort, which was constructed to explore the role of GA to predict the discontinuation of active cancer treatment [9]. This cohort consisted of patients with metastatic cancer with various solid cancer types, not hematologic malignancies, who received palliative first-line chemotherapy from October 2005 to March 2012. All patients received GA by trained geriatric nurse within 7 days before starting first-line chemotherapy.

### The selection of items from each domain of GA

In GA, functional status was evaluated using the activities of daily living (ADL, Barthel) and instrument activities of daily living (IADL, Lawton and Brody’s index) scores [21, 22]. Ten items of ADL consist of grooming, bathing, eating, dressing, toilet use, fecal and urinary continence, ascending and descending stairs, walking on a corridor. IADL consists of five items for men, including the ability to use the telephone, shopping, travel via car or public transportation, medication use, and ability to handle finances. For women, three more items including the ability to prepare food, to do laundry, and to do housekeeping were included. At least one dependency in ADL or IADL was categorized as ADL-dependent or IADL-dependent, respectively. Timed Get Up and Go test (TGUG) greater than 20 seconds were regarded as impaired mobility [23]. Cognitive function was evaluated using Mini-Mental Status Examination in the Korean version of the Consortium to Establish a Registry for Alzheimer's disease Assessment Packet (MMSE-KC), which was divided into severe cognitive impairment (scores ≤16) and mild cognitive impairment (scores 17−24) [24]. For depression, Short-Form Geriatric Depression Scale (SGDS) scores of 5 to 9 and of 10 or more indicated mild depression and severe depression, respectively, (ranging from 0 to 15) [25]. In terms of nutritional status, the Mini Nutritional Assessment (MNA) score less than 17 and between 17 and 23.5 indicated malnutrition and a risk for malnutrition, respectively [26]. Comorbidity was measured using the Charlson’s Comorbidity Index and was divided into low (0 points), medium (1–2 points), high (3–4 points), and very high (≥5 points) according to the original weighting system (e.g. AIDS is 6 points) [27]. Polypharmacy was evaluated based on numbers of taking drugs. Polypharmacy was descriptive and quantitative. However, impairment of polypharmacy was not defined.

All items within each domain were evaluated to predict the mild or severe impairment of each domain. SE, SP, PPV, and NPV were calculated to select the representative item for each domain. If a domain contained mild and severe impairment category, the mean value of SE or SP in each category were used to select the representative item of that domain. In the process of selection, items with SE >90% were identified first. If at least 2 items with SE >90% were not identified in a specific domain or items with SE >90% were not considered feasible for or applicable to cancer patients, additional items with SE >80% were identified. Of items with high sensitivity, 2 items with the highest balanced accuracy ((SE + SP)/2) were selected in each domain. The final items from each domain were rearranged by a panel of oncologists and geriatricians from the Korean Cancer Study Group (KCSG) Geriatric Oncology Working Party to evenly encompass most domains and also to be feasible for cancer patients in an outpatient setting.

### Cut-off value and internal/external validation of the novel screening tool

Abnormal GA was defined as deficits in at least 2 out of 6 domains, including ADL, IADL, SGDS, MNA, TGUG, and MMSE [19]. A cut-off value of the novel screening tool was decided using the maximal area under curve (AUC) in a receiver operating characteristic (ROC) curve. Because KG-7 is screening tool, high SE and NPV were considered with priority in selecting final questions. In a development cohort, the performance of KG-7 score was compared with that of G-8 score, using AUC. In the development cohort, internal validation was performed. External validation was done in a validation cancer cohort. G-8 score was calculated from MAN and age.

### Statistical analysis

The SE, SP, PPV, and NPV of each item for detecting impairment in each domain were calculated using a cross table. OS was measured from the date of GA to the last follow-up or any cause of death. The probability of OS was calculated using Kaplan-Meier survival analysis with log-rank significance tests. Hazard ratios (HR) were calculated using Cox regression hazard models. All analyses were performed using PASW Statistics 18 (SPSS Inc., Chicago, IL).

### Ethics statement

This study was approved by the Institutional Review Board and Independent Ethics Committee (IRB/IEC) of the Seoul National University Bundang Hospital (B-1310/222-111). IRB/IEC approved of waiving informed consents by reasons that this study would be conducted retrospectively, data collection would not harm the patient, and researchers would protect private information. To protect the private information, the patients were coded as serial number in the study. After the analysis of study, the private data were removed.

## Results

### Baseline characteristics of the development cohort

A total of 1284 patients who received GA were identified from the hospital registry in the development cohort. GA was performed in 55.8% and 44.2% of patients in inpatient (hospitalized) and outpatient clinics, respectively. Detailed baseline characteristics are presented in Table 1. The median age was 77 y (range 58−101 y). Patients ≥70 and ≥80 years of age comprised 86.2% and 35.7% of the development cohort, respectively. Medium risk, high risk, and very high risk measured by Charlson’s Comorbidity Index were 46.4%, 13.1%, and 10.0%, respectively. Most patients lived with family members (81.9%). Abnormal GA (defined as impairment in at least 2 domains) was documented in 763 patients (71.4%).

**Table 1:** Baseline characteristics including geriatric assessment (N = 1284).

Variable	N (%)
Median age (range)	77 (58–101)
≤59	2 (0.2%)
60–69	175 (13.6%)
70–79	649 (50.5%)
80–89	402 (31.3%)
≥90	56 (4.4%)
Sex	
Male	490 (38.2%)
Female	794 (61.8%)
Comorbidity (Charlson risk index)	
Low (0 points)	391 (30.5%)
Medium (1–2 points)	596 (46.4%)
High (3–4 points)	168 (13.1%)
Very high (≥5 points)	129 (10.0%)
Live with family member	
Yes	1052 (81.9%)
No	222 (17.3%
Missing	10 (0.8%)
Polypharmacy	
0–2	234 (18.2%)
3–4	211 (16.4%)
5-	776 (60.4%)
Missing	63 (4.9%)
Activities of daily living	
100 (independent)	652 (50.8%)
91–99	163 (12.7%)
76–90	102 (7.9%)
51–75	82 (6.4%)
-50	285 (22.2%)
Instrumental activities of daily living	
Independent	499 (38.9%)
Dependent	779 (60.7%)
Not capable	6 (0.4%)
Mini-mental status examination	
Intact (25–30)	480 (37.4%)
Mild impairment (17–24)	427 (33.3%)
Severe impairment (≤16, including not capable)	377 (29.4%)
Short form geriatric depression scale	
≥10 (severe depression)	206 (16.0%
10> ≥5 (mild depression)	327 (25.5%)
<5	579 (45.1%)
Not capable	172 (13.4%)
Mini nutritional assessment	
Normal (≥24)	355 (27.6%)
Risk of malnutrition (17≤ <24)	444 (34.6%)
Malnutrition (<17)	468 (36.4%)
Not capable	17 (1.3%)
Timed get up and go	
≤20	667 (51.9%)
>20 (including not capable)	558 (43.5%)
Missing	59 (4.6%)

### Selection process of items representing each GA domain

**Fig 1:**
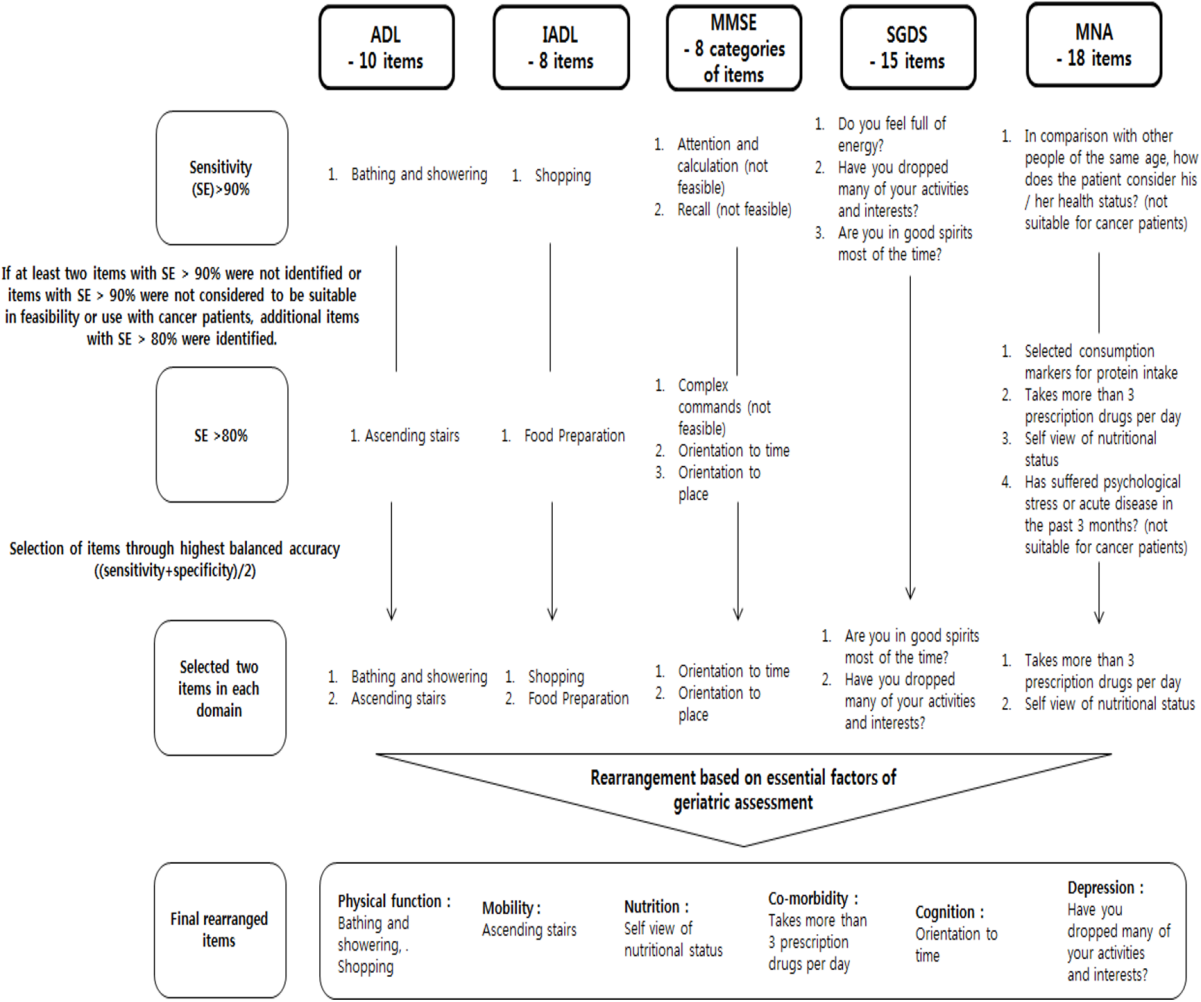
Selection process of representative items in each domain.

Each item of a GA domain was evaluated for SE and SP to detect impairment of the corresponding domain. The selection process is presented in Fig 1. Only “bathing and showering” showed a SE >90% (94.2%) for detecting impairment of ADL, so items with SE >80% were then selected. “Ascending stairs” was identified with a SE of 85.2%. Therefore, the items “bathing and showering” and “ascending stairs” were selected as representative items for the ADL domain. The screening values of each item for detecting impairment of ADL are listed in S1 Table. The SE, SP, PPV, and NPV of “shopping” for impairment of IADL were 91.1%, 100.0%, 100.0%, and 87.8%, respectively; the corresponding values were 89.8%, 100.0%, 100.0%, and 83.6% for “food preparation” (S2 Table). No other IADL items showed SE >80%. Therefore, “shopping” and “food preparation” were selected as representative items for IADL.

In the MMSE, “attention and calculation” and “recall” demonstrated mean SE >90% in detecting mild and severe cognitive impairment. The SE, SP, PPV, and NPV for mild cognitive impairment (or severe cognitive impairment) were 96.2% (100.0%), 64.8% (37.4%), 80.8% (35.6%), and 91.7% (100.0%), respectively, for “attention and calculation” and 92.2% (97.5%), 33.1% (23.0%), 67.0% (28.6%), and 74.1% (96.7%), respectively, for “recall” (S3 Table). However, these items were excluded because they were not feasible in an outpatient setting. Likewise, “complex commands”, “orientation of time”, and “orientation of place” were identified with a mean SE >80%, but “complex commands” was also excluded due to low feasibility. Therefore, the item categories of “orientation of time” and “orientation of place” were selected as representative items for mild and severe cognitive impairment. The SGDS items “Have you dropped many of your activities and interests?”, “Do you feel full of energy?”, and “Are you in good spirits most of the time?” had a mean SE >90% for mild and severe depression. The balanced accuracy of the SGDS items “Have you dropped many of your activities and interests?”, “Do you feel full of energy?”, and “Are you in good spirits most of the time?” was 75.2%, 77.0%, and 55.2%, respectively. We chose “Have you dropped many of your activities and interests?” and “Do you feel full of energy?” with higher balanced accuracy.

Regarding the MNA, the item “In comparison with other people of the same age, how do you consider your health status?” had a mean SE >90%, but this item was excluded due to not being suitable for cancer patients. The items “selected consumption markers for protein intake”, “taking more than 3 prescription drugs per day”, “self-view of nutritional status”, and “Has suffered psychological stress or acute disease in the past 3 months?” had a mean SE >80%. The item “Has suffered psychological stress or acute disease in the past 3 months?” was also excluded due to unsuitability in recently diagnosed cancer patients. Therefore, the MNA items “taking more than 3 prescription drugs per day” and “self-view of nutritional status” were identified through the highest balanced accuracy calculation. The detailed SE, SP, PPV, and NPV of each item in the SDGS and MNA are presented in S4 and S5 Tables, respectively.

### Korean Cancer Study Group Geriatric Score (KG)-7

According to the screening value of each item for assessing impairment of each corresponding domain, 2 items were selected from each domain. Selected items were rearranged to encompass most domains of GA without duplication, including physical function, mobility, nutrition, comorbidity, cognitive function, and depression, by a panel of oncologists and geriatricians from the Korean Cancer Study Group (KCSG) Geriatric Oncology Working Party. Finally, 7 questions were rearranged in the novel screening tool, the Korean Cancer Study Group Geriatric Score (KG)-7. The KG-7 consisted of 7 easy-to-answer questions evenly distributed to each domain of GA (Table 2; S6 Table for Korean version). KG-7 scores ranged from 0 to 7, and higher scores indicated better states.

**Table 2:** The Korean Cancer Study Group Geriatric Score (KG)-7.

1. Can you take a shower or bath without help?	Yes—1 point, No—0 point
2. Can you ascend the stairs without help?	Yes—1 point, No—0 point
3. Can you take care of all shopping needs independently?	Yes—1 point, No—0 point
4. How is the self-view of your nutritional status?	good—1 point, bad—0 point
5. Do you take more than 3 prescription drugs per day?	No—1 point, Yes—0 point
6. What year, month and day is this?	correct answer – 1 point, incorrect answer – 0 point
7. Have you dropped many of your activities and interests?	No—1 point, Yes—0 point
Total points	( ) /7 points

### Cut-off value and internal/external validation of the KG-7

In the ROC curve analysis, which showed an AUC of 0.93 (95% confidence interval (CI) 0.92−0.95), the cut-off value for detecting abnormal GA with the KG-7 was decided at ≤ 5 points, which showed a SE of 95.0% (95% CI 93.5–96.5) and SP of 59.2% (95% CI 53.7–64.7), to consider use for screening tool (S1 Fig).

The distribution of KG-7 scores in the development cohort is presented in Table 3. Based on the cut-off value of ≤ 5 points, the KG-7 showed a SE, SP, PPV, and NPV of 95.0% (95% CI 93.5–96.5), 59.2% (95% CI 53.7–64.7), 85.3% (95% CI 82.9–87.7), and 82.6% (95% CI 77.6–87.6), respectively, in internal validation for abnormal GA. KG-7 scores were normal (>5) in 20.5% of all patients, who could forgo full GA if the 2-step screening approach was adopted.

**Table 3:** The distribution of KG-7 score according to GA status in development cohort.

KG-7 score	0	1	2	3	4	5	6	7	Total
Normal CGA	0 (0.0%)	0 (0.0%)	0 (0.0%)	3 (2.5%)	20 (12.1%)	102 (53.7%)	135 (79.9%)	46 (92.0%)	306 (28.6%)
Abnormal CGA	121 (100.0%)	132 (100.0%)	124 (100.0%)	115 (97.5%)	145 (87.9%)	88 (46.3%)	34 (20.1%)	4 (8.0%)	763 (71.4%)
Total	121 (11.3%)	132 (12.3%)	124 (11.6%)	118 (11.0%)	165 (15.4%)	190 (17.8%)	169 (15.8%)	50 (4.7%)	1069 (100.0%)

The distribution of KG-7 scores in the validation cohort is shown in S7 Table. The AUC of the KG-7 for abnormal GA was 0.82 (95% CI 0.73−0.90; S2 Fig). In the validation cohort, the SE, SP, PPV, and NPV of the cut-off KG-7 score for abnormal GA (≤ 5 points) were 89.5% (95% CI 81.5–97.5), 48.6% (95% CI 32.5–64.7), 77.3% (95% CI 67.2–87.4), and 75.0% (95% CI 57.7–92.3), respectively. Likewise, 25.5% of all patients exhibited normal KG-7 scores. Additionally, the screening value of the KG-7 for severe impairment of at least 1 domain (ADL ≤75, IADL ≤3 (male) or ≤6 (female), MMSE <17, SGDS ≥10, MNA <17, TGUG ≥25 s), which was more important in the selection of fit cancer patients receiving standard treatment, was calculated. The AUC (0.91, 95% CI 0.85−0.97) of the KG-7 for severe impairment of GA was higher than for at least 2 two domain deficits. SE and NPV were much improved (97.6%, 95% CI 93.0–102.2 and 95.7%, 95% CI 87.4–104.0, respectively; S8 Table).

### The performance of KG-7 compared with G-8 in the prediction of abnormal GA

G-8 score was identified from MNA and age in the development cohort. The AUC of G-8 score for abnormal GA was 0.87 (95% CI 0.85–0.89, S3 Fig), which was lower than the AUC of KG-7 (0.93, 95% CI 0.92−0.95, S1 Fig). Based on the cut-off value of ≤ 14 points [20], the G-8 showed a SE, SP, PPV, and NPV of 97.5% (95% CI 96.5–98.5), 30.0% (95% CI 24.9–35.1), 81.1% (95% CI 78.8–83.4), and 79.7% (95% CI 72.4–87.0), respectively, in the prediction for abnormal GA. The SE of G-8 was slightly higher than that of KG-7. However, SP, PPV and NPV were lower than those of KG-7. Normal G-8 score (>14 points) was shown in only 9.2% of all patients. These patients who were screened not to receive full GA via the 2-step approach using G-8 were lower than KG-7 (20.5%).

### Overall survival according to KG-7 score

**Fig 2:**
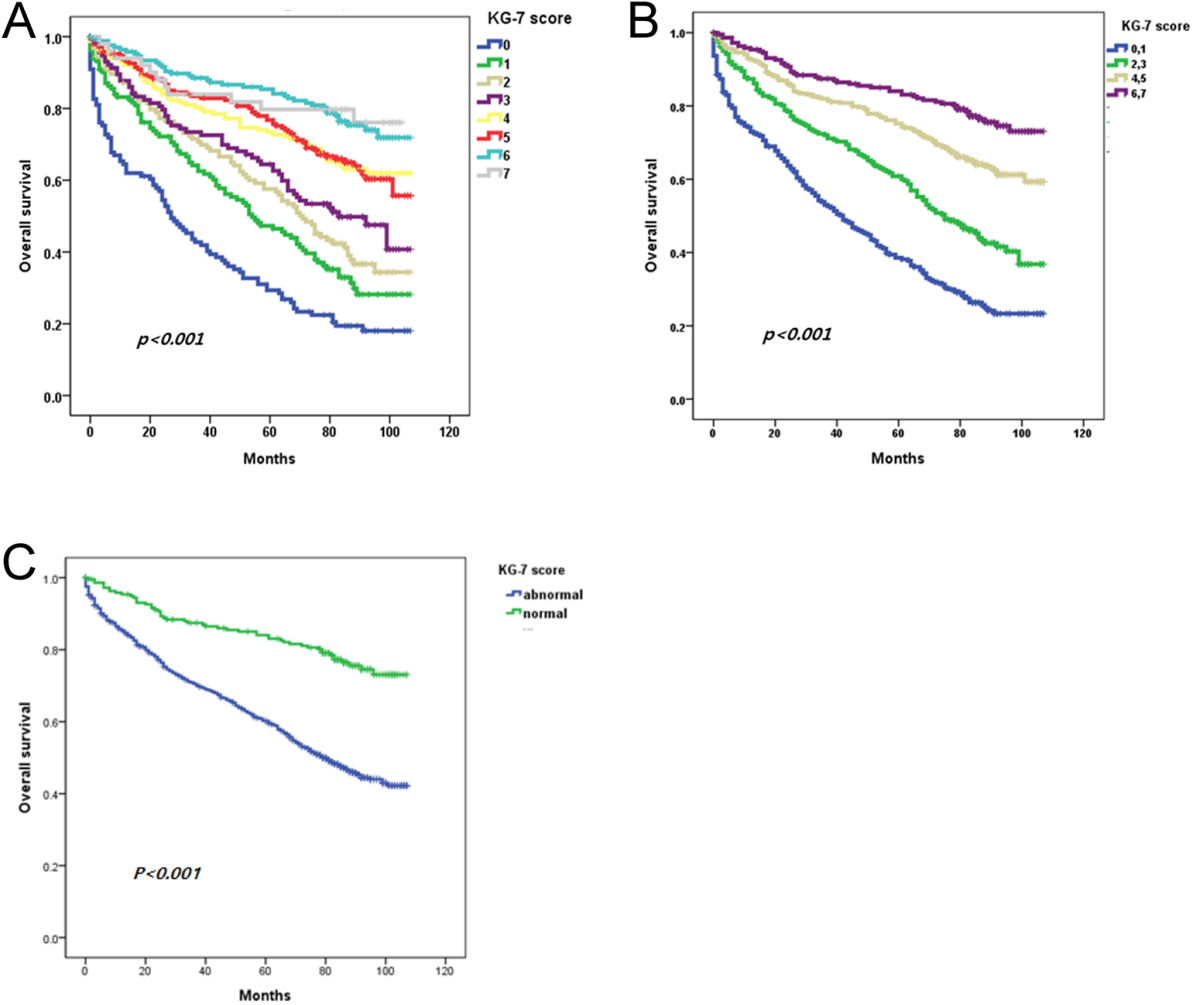
Overall survival according to KG-7 score in development cohort. (A) According to each KG-7 score. (B) According to categorized KG-7 score. (C) Between normal and abnormal KG-7 score.

KG-7 scores showed prognostic value for OS in the development cohort *(p <* 0.001; Fig 2A). When patients were categorized into 1 of 4 groups based on KG-7 score, patients with higher scores showed longer OS (*p* < 0.001; Fig 2B). Patients with normal KG-7 score (>5) had better survival than patients with abnormal KG-7 score (≤5; Fig 2C). The HR for survival between the groups with the lowest and highest KG-7 scores was 5.1 (95% CI 3.8−7.0, *p* < 0.001). In the validation cohort, KG-7 score could discriminate OS (*p* = 0.006; S4 Fig). Categorized groups also showed significantly different OS (*p* = 0.005). The HR for survival between patients with the highest versus lowest KG-7 scores was 3.7 (95% CI 1.6−8.8, *p* = 0.003).

## Discussion

We have developed a novel screening tool, the KG-7, suitable for an oncology clinic setting with high patient burden and low manpower resources to select patients who need full GA. The KG-7 consists of 7 items, each representative of essential GA domains, and was objectively derived from a large GA dataset. Internal validation in the development cohort and external validation in the validation cohort of older patients with cancer showed high screening value.

There are several noteworthy characteristics of the KG-7 compared to other screening tools. First of all, the KG-7 is based on large GA datasets, which have been considered the standard method in evaluating older patients. aCGA was produced by a similar method as our study [18]. However, physical function (ADL, IADL), cognition and geriatric depression as evaluating domains were limited in a relatively small population [18]. The G-8 and GFI were produced by experts and literature review [15, 20], and the proposition of a screening tool in G-8 was suggested [20]. However, the evidence for the selection of questions has rarely been reported in previous studies. Additionally, the items of the KG-7 were rearranged to encompass most items in GA evenly, with exception of fatigue, social status and comorbidity. The KG-7 consists of items distributed evenly across each essential domain of GA. However, previously developed screening tools do not reflect all domains of GA [19]. In the G-8, which was based on the MNA, nutritional status and weight loss are weighted higher than other domains [19, 20]. The psychosocial domain is dominant in the GFI, which enhanced psychosocial components. There are no questions related to nutrition in the aCGA [18, 19].

In previous systematic study, median SE of each frailty screening method for predicting frailty on GA was 68% in VES-13, 87% in G-8, 92% in TRST, 57% in GFI, and 51% in aCGA. NPV was approximately 60% [19]. KG-7 showed high SE and NPV (95.0%, 82.6% in development cohort, 89.5% and 75%, respectively in validation cohort) [19]. This value of KG-7 could be comparable with that of other screening tools. Moreover, in comparison with G-8 within the development cohort, the AUC of KG-7 score for abnormal GA was higher. Therefore, KG-7 could be used comparably with other screening tools if KG-7 would show the similar screening value in a prospective validation.

Notably, the KG-7 was developed considering the feasibility for and use with cancer patients. The G-8 and aCGA were also designed for older patients with cancer, but the VES-13 and TRST were produced for community members and visitors to the emergency room, respectively [16, 17]. The selection process of items for the KG-7 was based on the use with cancer patients. Therefore, factors relating to comorbidities were excluded because cancer was a major comorbidity in the study population. Items not suitable for cancer patients were also excluded from the KG-7, such as the MNA items “In comparison with other people of the same age, how do you consider your health status?” and “Has suffered psychological stress or acute disease in the past 3 months?”. The KG-7 also consists of all easy-to-answer questions. For feasibility, complex questions from cognitive function tests, including “attention and calculation”, “recall”, and “complex commands”, were excluded from the KG-7 although they showed high sensitivity. Finally, the KG-7 has prognostic value. In previous reports, GA predicted the early death or survival in older patients with cancer [7, 8]. Furthermore, the G-8 and TRST also reflected prognosis of older patients with cancer [14]. In concordance with previous studies, survival was significantly discriminated according to KG-7 scores.

In this study, abnormal GA was defined as deficits in at least 2 domains, a definition which was commonly used in previous studies [19]. However, the definition of abnormal GA varied across studies of the validation of screening tools. According to the definitions of abnormal GA, the rates of abnormal GA would be different, and screening values such as SE, SP, PPV, and NPV would also differ. In the present study, the screening value of the KG-7 for severe impairment of at least 1 domain was calculated additionally in a validation cohort of cancer patients. The AUC of ROC, SE, and NPV of the KG-7 were much improved in comparison to the screening value of the KG-7 for deficits in at least 2 domains. Therefore, the KG-7 would be useful when incorporated in patient selection for active chemotherapy in older patients with cancer.

There are some limitations to our study. First, the development cohort did not derive from cancer patients but was constructed from heterogeneous patients who received GA with various diagnoses, and only 12.2% of patients had a past medical history of cancer or current cancer. Also, the median age of the development cohort was 77 y, and approximately 14% of patients were less than 70 years old. We focused on the development of a screening tool to represent the entire GA. Therefore, patients with various diseases or patients less than 70 years in age were not excluded from this study. Moreover, because the phenotype of aging is more important than numeric age, patients who received GA despite relatively younger age (<70 y) were included in this study. Second, some specific domains of GA, such as social support, fatigue, and geriatric syndrome, were not considered in the KG-7. We strove to include as many GA domains as possible. However, the domains utilized in full GA are different across studies. Therefore, the KG-7 includes physical function, mobility, nutrition, comorbidity, cognition, and depression. In comparison to previous studies of GA [19], GA consisting of these domains could be considered enough to evaluate older patients with cancer. Third, the size of the validation cohort is very small. The validation cohort was used to identify a possibility of KG-7 as screening tool. Prospective validation is warranted.

In conclusions, we have developed a screening tool (the KG-7) with easy-to-answer questions and high performance objectively selected from a large dataset to represent each domain of GA with high SE and NPV. Moreover, the KG-7 can predict survival in older patients. A prospective validation study to test the usefulness of the KG-7 as a screening tool in older patients with cancer receiving first-line palliative chemotherapy is currently underway. Given the results of our studies, the KG-7 could be used effectively in countries with high patient burden and low resources to select patients in need of full GA and intervention.

## Supporting Information

S1 Fig ROC curve of KG-7 in development cohort. (DOCX)

S2 Fig ROC curve of KG-7 in validation cohort. (DOCX)

S3 Fig ROC curve of G-8 in development cohort. (DOCX)

S4 Fig Overall survival according to KG-7 score in validation cohort. (DOCX)

S1 Table The screening value of each item for impairment of ADL. (DOCX)

S2 Table The screening value of each item for impairment of IADL. (DOCX)

S3 Table The screening value of each item for impairment of MMSE-KC. (DOCX)

S4 Table The screening value of each item for impairment of SGDS. (DOCX)

S5 Table The screening value of each item for impairment of MNA. (DOCX)

S6 Table The Korean Cancer Study Group Geriatric Score (KG)-7 (Korean version). (DOCX)

S7 Table The distribution of KG-7 score according to GA status in validation cohort. (DOCX)

S8 Table The distribution of KG-7 score according to GA status in validation cohort (abnormal GA was defined as at least one severe form of domain). (DOCX)
