# Supplementary figures and images for: A Novel Geriatric Screening Tool in Older Patients with Cancer: The Korean Cancer Study Group Geriatric Score (KG)-7
Source: PLoS One. 2015 Sep 24;10(9):e0138304. doi: 10.1371/journal.pone.0138304 (PMC4581840; doi:10.1371/journal.pone.0138304)

S1 Fig. ROC curve of KG-7 in development cohort


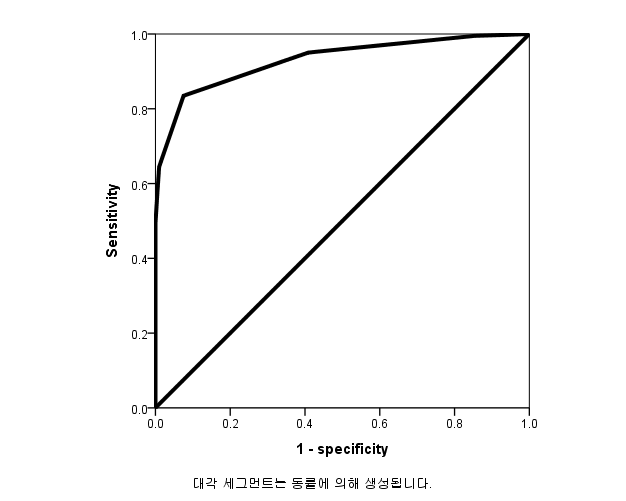

Supplement: S1 Fig — (DOCX) [file pone.0138304.s001.docx]

S2 Fig. ROC curve of KG-7 in validation cohort


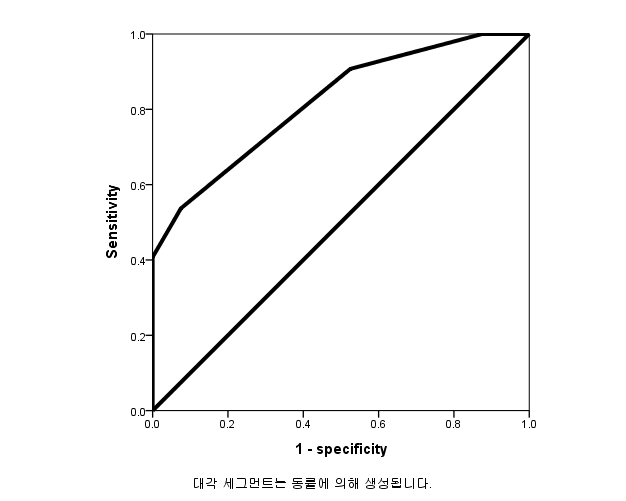

Supplement: S2 Fig — (DOCX) [file pone.0138304.s002.docx]

S3 Fig. ROC curve of G-8 in development cohort


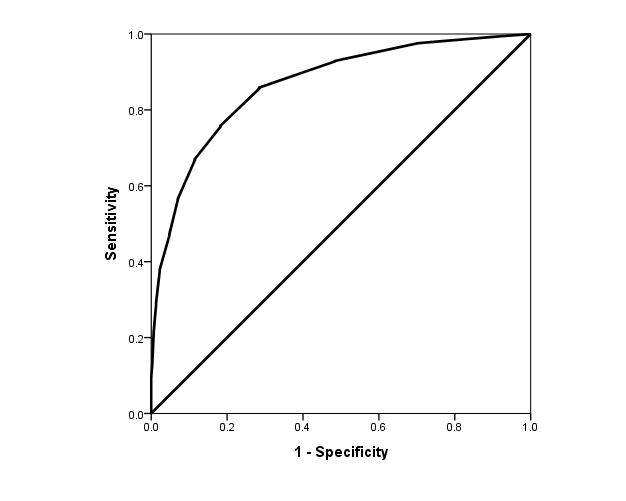

Supplement: S3 Fig — (DOCX) [file pone.0138304.s003.docx]

S4 Fig. Overall survival according to KG-7 score in validation cohort


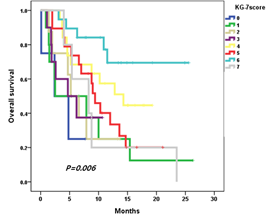

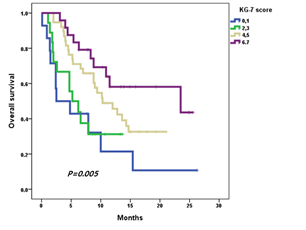

Supplement: S4 Fig — (DOCX) [file pone.0138304.s004.docx]
