# Supplementary material for: A Novel Geriatric Screening Tool in Older Patients with Cancer: The Korean Cancer Study Group Geriatric Score (KG)-7
Source: PLoS One. 2015 Sep 24;10(9):e0138304. doi: 10.1371/journal.pone.0138304 (PMC4581840; doi:10.1371/journal.pone.0138304)
Supplement: S1 Table — (DOCX) [file pone.0138304.s005.docx]

S1 Table. The screening value of each item for impairment of ADL

| ADL | Sensitivity | Specificity | Positive predictive value | Negative predictive value |
| --- | --- | --- | --- | --- |
| Personal hygiene and grooming | 65.0% | 100.0% | 100.0% | 74.7% |
| Bathing and showering | 92.4% | 100.0% | 100.0% | 93.1% |
| Eating and feeding | 55.5% | 100.0% | 100.0% | 70.0% |
| Toilet hygiene | 65.4% | 100.0% | 100.0% | 75.0% |
| Ascending stairs | 85.2% | 100.0% | 100.0% | 87.5% |
| Dressing | 68.8% | 100.0% | 100.0% | 76.8% |
| Bowel management | 57.5% | 100.0% | 100.0% | 70.9% |
| Bladder management | 60.4% | 100.0% | 100.0% | 72.4% |
| Transfer | 65.3% | 100.0% | 100.0% | 75.1% |
| Mobility | 62.3% | 100.0% | 100.0% | 78.2% |
