# Supplementary material for: A Novel Geriatric Screening Tool in Older Patients with Cancer: The Korean Cancer Study Group Geriatric Score (KG)-7
Source: PLoS One. 2015 Sep 24;10(9):e0138304. doi: 10.1371/journal.pone.0138304 (PMC4581840; doi:10.1371/journal.pone.0138304)
Supplement: S2 Table — (DOCX) [file pone.0138304.s006.docx]

S2 Table. The screening value of each item for impairment of IADL

| IADL | Sensitivity | Specificity | Positive predictive value | Negative predictive value |
| --- | --- | --- | --- | --- |
| Ability to use telephone | 31.7% | 100.0% | 100.0% | 48.4% |
| Shopping | 91.1% | 100.0% | 100.0% | 87.8% |
| Mode of Transportation | 76.5% | 100.0% | 100.0% | 73.2% |
| Responsibility for own medications | 55.7% | 100.0% | 100.0% | 59.5% |
| Ability to Handle Finances | 51.0% | 100.0% | 100.0% | 57.0% |
| Food Preparation | 89.8% | 100.0% | 100.0% | 83.6% |
| Housekeeping | 52.7% | 100.0% | 100.0% | 52.4% |
| Laundry | 63.2% | 100.0% | 100.0% | 58.7% |
