# Supplementary material for: A Novel Geriatric Screening Tool in Older Patients with Cancer: The Korean Cancer Study Group Geriatric Score (KG)-7
Source: PLoS One. 2015 Sep 24;10(9):e0138304. doi: 10.1371/journal.pone.0138304 (PMC4581840; doi:10.1371/journal.pone.0138304)
Supplement: S3 Table — (DOCX) [file pone.0138304.s007.docx]

S3 Table. The screening value of each item for impairment of MMSE-KC

| MMSE-KC | Sensitivity,% - for over mild cognitive impairment/severe cognitive impairment (mean value) | Specificity,%- for over mild cognitive impairment/severe cognitive impairment (mean value) | Positive predictive value,%- for over mild cognitive impairment/severe cognitive impairment (mean value) | Negative predictive value,%- for over mild cognitive impairment/severe cognitive impairment (mean value) |
| --- | --- | --- | --- | --- |
| Orientation to time | 72.8/99.3 (86.1) | 93.3/70.9 (82.1) | 94.3/52.8 (73.6) | 69.5/99.7 (84.6) |
| Orientation to place | 73.6/98.0 (85.8) | 80.4/63.0 (71.7) | 85.0/46.4 (65.7) | 66.9/99.0 (83.0) |
| Registration | 27.5/50.0 (38.8) | 96.0/92.3 (94.2) | 91.2/67.7 (79.5) | 46.7/85.0 (65.9) |
| Attention and calculation | 96.2/100.0 (98.1) | 64.8/37.4 (51.1) | 80.8/35.6 (58.2) | 91.7/100.0 (95.9) |
| Recall | 92.2/97.5 (94.6) | 33.1/23.0 (28.1) | 67.0/28.6 (47.8) | 74.1/96.7 (85.4) |
| Language | 7.7/17.5 (12.6) | 99.8/99.4 (99.6) | 98.2/91.1 (94.7) | 42.0/78.9 (60.5) |
| Repetition | 21.7/44.0 (32.9) | 99.2/96.6 (97.9) | 97.5/80.6 (89.1) | 45.8/84.2 (65.0) |
| Complex commands | 80.9/97.6 (89.3) | 76.7/54.9 (65.8) | 83.8/40.9 (62.4) | 72.9/98.6 (85.8) |
