# Supplementary material for: A Novel Geriatric Screening Tool in Older Patients with Cancer: The Korean Cancer Study Group Geriatric Score (KG)-7
Source: PLoS One. 2015 Sep 24;10(9):e0138304. doi: 10.1371/journal.pone.0138304 (PMC4581840; doi:10.1371/journal.pone.0138304)
Supplement: S4 Table — (DOCX) [file pone.0138304.s008.docx]

S4 Table. The screening value of each item for impairment of SGDS

| SGDS | Sensitivity,%- for over mild depression/severe depression (mean value) | Specificity,%- for over mild depression/severe depression (mean value) | Positive predictive value,%- for over mild depression/severe depression (mean value) | Negative predictive value,%- for over mild depression/severe depression (mean value) |
| --- | --- | --- | --- | --- |
| Are you basically satisfied with your life? | 52.0/79.5 (65.8) | 92.9/83.1 (88.0) | 87.0/51.7 (69.4) | 67.9/94.7 (81.3) |
| Have you dropped many of your activities and interests? | 90.6/98.0 (94.3) | 65.2/46.8 (56.0) | 70.5/29.5 (50.0) | 88.3/99.1 (93.7) |
| Do you feel that your life is empty? | 65.8/92.7 (79.3) | 92.7/77.9 (85.3) | 89.2/49.2 (69.2) | 74.7/97.9 (86.3) |
| Do you often get bored? | 73.5/89.8 (81.7) | 90.9/71.4 (81.2) | 88.2/41.8 (65.0) | 78.8/96.8 (87.8) |
| Are you in good spirits most of the time? | 86.8/96.1 (91.5) | 72.0/52.9 (62.5) | 74.2/32.0 (53.1) | 85.4/98.3 (91.9) |
| Are you afraid that something bad is going to happen to you? | 50.2/60.3 (55.3) | 85.4/74.8 (80.1) | 75.8/35.0 (55.4) | 65.2/89.4 (77.3) |
| Do you feel happy most of the time? | 60.7/87.6 (74.2) | 96.5/82.0 (89.3) | 94.1/52.4 (73.3) | 72.9/96.7 (84.8) |
| Do you often feel helpless? | 74.0/97.6 (85.8) | 94.6/75.4 (85.0) | 92.6/47.6 (70.1) | 79.9/99.3 (89.6) |
| Do you prefer to stay at home, rather than going out and doing new things? | 53.2/69.8 (61.5) | 85.2/75.3 (80.3) | 76.5/39.0 (57.8) | 66.8/91.7 (79.3) |
| Do you feel you have more problems with memory than most people? | 33.3/46.8 (40.1) | 92.1/86.1 (89.1) | 79.5/43.6 (61.6) | 60.0/87.6 (73.8) |
| Do you think it is wonderful to be alive? | 59.8/89.2 (74.5) | 96.9/83.1 (90.0) | 94.6/54.5 (74.6) | 72.4/97.1 (84.8) |
| Do you feel pretty worthless the way you are now? | 62.5/94.2 (78.4) | 95.5/81.7 (88.6) | 92.7/54.2 (73.5) | 73.3/98.4 (85.9) |
| Do you feel full of energy? | 97.7/99.0 (98.4) | 14.0/10.0 (12) | 51.4/20.1 (35.8) | 87.0/97.8 (92.4) |
| Do you feel that your situation is hopeless? | 29.6/59.7 (44.7) | 99.8/96.0 (97.9) | 99.4/77.4 (88.4) | 60.4/91.2 (75.8) |
| Do you think that most people are better off than you are? | 30.8/57.8 (44.3) | 97.7/93.5 (95.6) | 92.7/67.2 (80.0) | 60.2/90.6 (75.4) |
