# Supplementary material for: A Novel Geriatric Screening Tool in Older Patients with Cancer: The Korean Cancer Study Group Geriatric Score (KG)-7
Source: PLoS One. 2015 Sep 24;10(9):e0138304. doi: 10.1371/journal.pone.0138304 (PMC4581840; doi:10.1371/journal.pone.0138304)
Supplement: S5 Table — (DOCX) [file pone.0138304.s009.docx]

S5 Table. The screening value of each item for impairment of MNA

| MNA | Sensitivity,%- for over a risk for malnutrition/malnutrition (mean value) | Specificity,%- for over a risk for malnutrition/malnutrition (mean value) | Positive predictive value,%- for over a risk for malnutrition/malnutrition (mean value) | Negative predictive value,%- for over a risk for malnutrition/malnutrition (mean value) |
| --- | --- | --- | --- | --- |
| Body Mass Index | 61.6/78.0 (69.8) | 80.2/66.5 (73.4) | 88.7/57.1 (72.9) | 45.3/84.1 (64.7) |
| Has food intake declined over the past 3 months due to loss  of appetite, digestive problems, chewing or swallowing  difficulties? | 72.8/87.0 (79.9) | 87.6/62.4 (75.0) | 93.8/57.6 (75.7) | 55.6/89.1 (72.4) |
| Has suffered psychological stress or acute disease in the  past 3 months? | 76.3/88.0 (82.2) | 81.9/56.4 (69.2) | 91.5/54.2 (72.9) | 57.3/88.9 (73.1) |
| Weight loss during the last 3 months | 66.9/85.0 (76.0) | 87.6/68.0 (77.8) | 93.3/60.9 (77.1) | 50.7/88.6 (69.7) |
| Mobility | 53.2/77.9 (65.6) | 93.0/81.7 (87.4) | 95.1/71.4 (83.3) | 43.7/86.4 (65.1) |
| Neuropsychological problems | 44.0/58.7 (51.4) | 88.4/79.0 (83.7) | 90.7/62.1 (76.4) | 38.1/76.5 (57.3) |
| Lives independently (not in nursing home or hospital) | 42.9/67.5 (55.2) | 96.9/89.1 (93.0) | 97.3/78.3 (87.8) | 39.7/82.5 (61.1) |
| Takes more than 3 prescription drugs per day | 82.5/85.4 (84.0) | 29.8/24.6 (27.2) | 75.4/39.9 (57.7) | 39.5/74.1 (56.8) |
| Pressure sores or skin ulcers | 9.0/13.9 (11.5) | 98.3/97.1 (97.7) | 93.2/73.9 (83.6) | 29.6/65.8 (47.7) |
| How many full meals does the patient eat daily? | 21.8/31.0 (26.4) | 93.8/90.5 (92.2) | 90.0/65.5 (77.8) | 31.8/69.2 (50.5) |
| Selected consumption markers for protein intake | 87.0/88.6 (87.8) | 17.5/15.9 (16.7) | 73.1/38.3 (55.7) | 34.3/70.2 (52.3) |
| Consumes two or more servings of fruit or vegetables  per day? | 34.3/49.5 (41.9) | 94.1/87.2 (90.7) | 93.7/69.3 (81.5) | 35.8/74.7 (55.3) |
| How much fluid (water, juice, coffee, tea, milk...) is  consumed per day? | 68.1/77.4 (72.8) | 55.8/48.0 (51.9) | 79.8/46.6 (63.2) | 40.6/78.4 (59.5) |
| Mode of feeding | 30.7/51.7 (41.2) | 98.9/94.7 (96.8) | 98.6/85.2 (91.9) | 35.7/77.0 (56.4) |
| Self view of nutritional status | 75.4/92.3 (83.9) | 92.4/64.9 (78.7) | 96.2/60.8 (78.5) | 59.5/93.5 (76.5) |
| In comparison with other people of the same age, how does  the patient consider his / her health status? | 87.2/97.2 (92.2) | 49.9/35.2 (42.5) | 81.7/46.8 (64.3) | 60.2/95.6 (77.9) |
| Mid-arm circumference | 35.1/54.4 (44.8) | 98.3/91.0 (94.7) | 98.2/77.8 (88.0) | 37.1/77.4 (57.3) |
| Calf circumference | 60.8/81.5 (71.2) | 89.8/73.7 (81.8) | 93.9/64.3 (79.1) | 47.2/87.2 (67.2) |
