# Supplementary material for: A Novel Geriatric Screening Tool in Older Patients with Cancer: The Korean Cancer Study Group Geriatric Score (KG)-7
Source: PLoS One. 2015 Sep 24;10(9):e0138304. doi: 10.1371/journal.pone.0138304 (PMC4581840; doi:10.1371/journal.pone.0138304)
Supplement: S6 Table — (DOCX) [file pone.0138304.s010.docx]

S6 Table. The Korean Cancer Study Group Geriatric Score (KG)-7 (Korean version)

| 1. 도움 없이 혼자서 목욕을 하실 수 있습니까? | 예 -1점, 아니오- 0점 |
| --- | --- |
| 2. 도움 없이 혼자서 계단을 오를 수 있습니까? | 예 -1점, 아니오- 0점 |
| 3. 필요한 물건은 모두 혼자서 구입할 수 있습니까? | 예 -1점, 아니오- 0점 |
| 4. 본인의 영양 상태를 스스로 평가하시면 어떻습니까? | 양호- 1점, 불량- 0 점 |
| 5. 현재 매일 3가지 이상의 약물을 복용하고 있습니까? | 없다- 1점, 있다- 0점 |
| 6. 오늘이 몇 년도 며칠 입니까? | 맞춤- 1점, 못 맞춤 -0점 |
| 7. 활동이나 관심거리가 많이 줄었습니까? | 아니오-1점, 예-0점 |
| 총점 | ( )/ 7점 |
