# Supplementary material for: A Novel Geriatric Screening Tool in Older Patients with Cancer: The Korean Cancer Study Group Geriatric Score (KG)-7
Source: PLoS One. 2015 Sep 24;10(9):e0138304. doi: 10.1371/journal.pone.0138304 (PMC4581840; doi:10.1371/journal.pone.0138304)
Supplement: S7 Table — (DOCX) [file pone.0138304.s011.docx]

S7 Table. The distribution of KG-7 score according to GA status in validation cohort

| KG-7 score | 0 | 1 | 2 | 3 | 4 | 5 | 6 | 7 | Total |
| --- | --- | --- | --- | --- | --- | --- | --- | --- | --- |
| Normal CGA | 0 | 0 | 0 | 2 | 9 | 8 | 13 | 5 | 37 |
|  | 0.0% | 0.0% | 0.0% | 20.0% | 47.4% | 42.1% | 68.4% | 100.0% | 39.4% |
| Abnormal CGA | 4 | 10 | 8 | 8 | 10 | 11 | 6 | 0 | 57 |
|  | 100.0% | 100.0% | 100.0% | 80.0% | 52.6% | 57.9% | 31.6% | 0.0% | 60.6% |
| Total | 4 | 10 | 8 | 10 | 19 | 19 | 19 | 5 | 94 |
|  | 4.3% | 10.6% | 8.5% | 10.6% | 20.2% | 20.2% | 20.2% | 5.3% | 100.0% |
