# Supplementary material for: A Novel Geriatric Screening Tool in Older Patients with Cancer: The Korean Cancer Study Group Geriatric Score (KG)-7
Source: PLoS One. 2015 Sep 24;10(9):e0138304. doi: 10.1371/journal.pone.0138304 (PMC4581840; doi:10.1371/journal.pone.0138304)
Supplement: S8 Table — (DOCX) [file pone.0138304.s012.docx]

S8 Table. The distribution of KG-7 score according to GA status in validation cohort (abnormal GA was defined as at least one severe form of domain)

| KG-7 | 0 | 1 | 2 | 3 | 4 | 5 | 6 | 7 | Total |
| --- | --- | --- | --- | --- | --- | --- | --- | --- | --- |
| Normal CGA | 0 | 0 | 0 | 3 | 10 | 16 | 18 | 4 | 51 |
|  | 0.0% | 0.0% | 0.0% | 30.0% | 52.6% | 84.2% | 94.7% | 100.0% | 54.8% |
| at least one severe impairment of domain | 4 | 10 | 8 | 7 | 9 | 3 | 1 | 0 | 42 |
|  | 100.0% | 100.0% | 100.0% | 70.0% | 47.4% | 15.8% | 5.3% | 0.0% | 45.2% |
| Total | 4 | 10 | 8 | 10 | 19 | 19 | 19 | 4 | 93 |
|  | 4.3% | 10.8% | 8.6% | 10.8% | 20.4% | 20.4% | 20.4% | 4.3% | 100.0% |
